# Sensory recalibration integrates information from the immediate and the cumulative past

**DOI:** 10.1038/srep12739

**Published:** 2015-08-04

**Authors:** Patrick Bruns, Brigitte Röder

**Affiliations:** 1 Biological Psychology and Neuropsychology, University of Hamburg, Hamburg, Germany

## Abstract

Vision usually provides the most accurate and reliable information about the location of objects in our environment, and thus serves as a reference for recalibrating auditory spatial maps. Recent studies have shown that recalibration does not require accumulated evidence of cross-modal mismatch to be triggered, but occurs as soon as after one single exposure. Here we tested whether instantaneous recalibration and recalibration based on accumulated evidence represent the same underlying learning mechanism or involve distinct neural systems. Participants had to localize two sounds, a low- and a high-frequency tone, which were paired with opposite directions of audiovisual spatial mismatch (leftward vs. rightward). In accordance with the cumulative stimulus history, localization in unimodal auditory trials was shifted in opposite directions for the two sound frequencies. On a trial-by-trial basis, however, frequency-specific recalibration was reduced when preceded by an audiovisual stimulus with a different sound frequency and direction of spatial mismatch. Thus, the immediate past invoked an instantaneous frequency-invariant recalibration, while the cumulative past invoked changes in frequency-specific spatial maps. These findings suggest that distinct recalibration mechanisms operating at different timescales jointly determine sound localization behavior.

Cross-modal recalibration guarantees a continuous alignment of our senses and thus allows for a coherent representation of the outside world. Recalibration comes into play whenever the sensory environment changes, for example, when we move from outside into a room, or whenever the sensory organs change, for example, when the sensory capacities decline as when we age^1^,^2^. The precise mechanisms of cross-modal recalibration are still not understood. Indeed there is a debate of whether representations in early sensory cortex^3^,^4^,^5^,^6^ or rather in multisensory association cortex^7^,^8^,^9^ are adjusted. Moreover, traditionally it has been assumed that sensory recalibration is only initiated after cross-modal mismatch has prevailed for a longer duration^10^, while more recent studies have suggested that recalibration is initiated instantaneously after a single exposure to a misaligned cross-modal stimulus^11^,^12^.

The present study used the ventriloquism aftereffect, in which repeated exposure to spatially misaligned audiovisual stimuli induces a subsequent shift in unisensory sound localization^13^, to assess the mechanisms of cross-modal spatial recalibration. Based on the psychophysical finding that the ventriloquism aftereffect is specific for the sound frequency used during recalibration, it has been argued that spatial representations in tonotopically-organized auditory areas are cross-modally adjusted^3^,^4^,^5^,^6^. This is in line with the finding that the ventriloquism aftereffect is associated with a modulation of an early auditory evoked brain potential around 100 ms after stimulus onset^14^, which likely has generators in auditory sensory cortex^15^,^16^. By contrast, the immediate visual bias of auditory localization observed during audiovisual stimulation (the online ventriloquist effect) seems to occur later in the processing hierarchy^17^,^18^, possibly involving feedback influences from multisensory areas on secondary sensory cortices^17^,^19^,^20^,^21^. We, therefore, hypothesized that a recalibration of early sensory processing requires accumulated evidence of a consistent audiovisual spatial misalignment, while instantaneous recalibration following a single exposure to a new mismatch of existing cross-modal correspondences is based on the same feedback mechanism that is active during the preceding audiovisual stimulation.

In order to test these assumptions, we reassessed the sound frequency-specificity of the ventriloquism aftereffect with a new paradigm. It was tested whether observers are able to simultaneously adapt to two opposing spatial relationships (i.e., aftereffects induced in opposite directions) when each is associated with one of two different sound frequencies. More important, the ventriloquism aftereffect was assessed on a trial-by-trial basis, allowing us to test whether the sound frequency-specificity of the ventriloquism aftereffect, which would suggest a recalibration of spatial representations in early auditory cortex, varies as a function of the cumulative and immediate stimulus history. In particular, we expected that if the cumulative stimulus history results in a frequency-specific recalibration, localization responses should systematically differ between the leftward-adapted and the rightward-adapted sound frequency. An additional modulation of sound localization, depending on the direction of audiovisual spatial mismatch in the directly preceding adaptation trial, would only be expected if the immediate recalibration effect is frequency-unspecific. If only the cumulative past, but not the immediate past, results in a frequency-specific recalibration, a parallel adjustment of two distinct spatial representations would be suggested.

## Results

Participants performed a sound localization task, in which they indicated the perceived location of 750 Hz and 3000 Hz tones. The sounds were presented from one of six different azimuthal positions spanning ±22.5°, either alone (test trials to assess the ventriloquism aftereffect), or together with synchronous visual stimuli that were displaced by 13.5° to the left or to the right of the sound source (adaptation trials). Importantly, the two sound frequencies were associated with a fixed audiovisual spatial relationship: For half of the participants, the 750 Hz tone was always presented with a visual stimulus displaced to the left and the 3000 Hz tone with a visual stimulus displaced to the right (750L/3000R), and vice versa for the other half of the participants (750R/3000L).

### Localization Responses in Audiovisual Adaptation Trials

**Figure 1:**
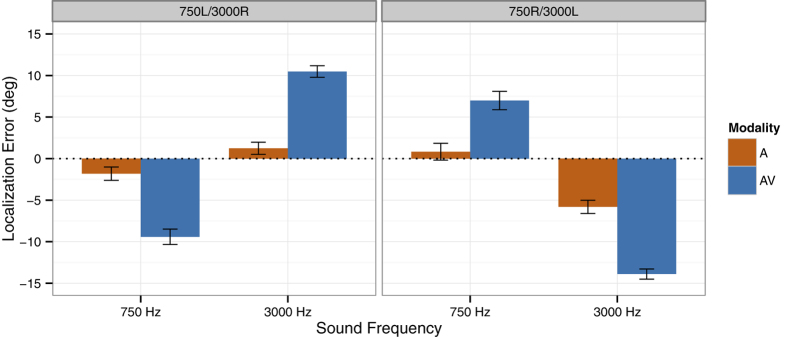
Localization responses in audiovisual (AV) and auditory-only (A) trials. Mean constant errors (i.e., the mean deviation of the spatial responses from the location of the sound source) across all loudspeaker locations are shown separately for the 750 Hz and 3000 Hz tones. The left panel shows the data for the subgroup of participants (*n *= 15) in which the 750 Hz tone was paired with a visual stimulus to the left and the 3000 Hz tone with a visual stimulus to the right (750L/3000R). Data for the subgroup of participants (*n *= 15) in which the 750 Hz tone was paired with a visual stimulus to the right and the 3000 Hz tone with a visual stimulus to the left (750R/3000L) are shown in the right panel. Negative values indicate constant errors to the left of the true stimulus location, and positive values indicate constant errors to the right. Error bars denote the *SEM*.

In accordance with the audiovisual spatial mismatch, sound localization responses for adaptation trials were shifted toward the location of the concurrent visual stimuli, as indicated by a highly significant interaction of Group (750L/3000R or 750R/3000L) and Sound Frequency (750 Hz or 3000 Hz), *F*(1, 28) = 156.47, *p *< .001 (see Fig. 1). In both groups, localization responses in audiovisual adaptation trials differed significantly between the two sound frequencies (both *p*s < .001, Bonferroni-corrected), while the size of this difference did not differ between the 750L/3000R group (*M *= 19.9°, *SEM *= 2.3°) and the 750R/3000L group (*M *= 20.9°, *SEM *= 2.3°), *t *< 1. This visual bias of auditory localization reflects the well-known ventriloquism effect^22^,^23^,^24^,^25^ and verifies that the visual stimuli indeed changed the perceived sound location in audiovisual adaptation trials.

### Cumulative and Immediate Recalibration Effects

Crucially, auditory localization was altered for the unimodal auditory test trials as well (see Fig. 1). The novel finding here is that a ventriloquism aftereffect emerged in opposite directions for the two different sound frequencies at the same time, as indicated by a highly significant interaction of Group (750L/3000R or 750R/3000L) and Sound Frequency (750 Hz or 3000 Hz), *F*(1, 28) = 19.91, *p *< .001. Post-hoc *t* tests showed that, in both groups, localization responses in unimodal auditory test trials differed significantly between the leftward-adapted and the rightward-adapted sound frequency (both *p*s < .05, Bonferroni-corrected). The size of this aftereffect did not differ significantly between the 750L/3000R group (*M *= 3.1°, *SEM *= 1.0°) and the 750R/3000L group (*M *= 6.6°, *SEM *= 1.9°), *t*(28) = 1.64, *p *= .112. There was no significant main effect or interaction involving Block in the overall ANOVA (all *p*s ≥ .266), suggesting that the size of the aftereffect did not change between the three experimental blocks.

**Figure 2:**
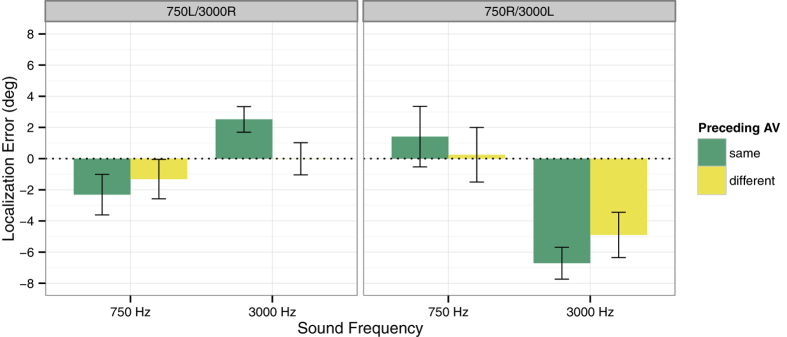
Localization responses in auditory-only trials following an audiovisual (AV) trial with the same or different sound frequency. Mean constant errors (i.e., the mean deviation of the spatial responses from the location of the sound source) across all loudspeaker locations are shown separately for the 750 Hz and 3000 Hz tones. The left panel shows the data for the subgroup of participants (*n *= 15) in which the 750 Hz tone was paired with a visual stimulus to the left and the 3000 Hz tone with a visual stimulus to the right (750L/3000R). Data for the subgroup of participants (*n *= 15) in which the 750 Hz tone was paired with a visual stimulus to the right and the 3000 Hz tone with a visual stimulus to the left (750R/3000L) are shown in the right panel. Negative values indicate constant errors to the left of the true stimulus location, and positive values indicate constant errors to the right. Error bars denote the *SEM*.

To test whether adaptation to the cumulative stimulus history was further modulated by the immediate stimulus history, we analysed localization responses in unimodal auditory test trials as a function of whether or not the immediately preceding audiovisual adaptation trial comprised the same sound frequency as the auditory test trial. We found that sound localization responses in unimodal auditory test trials were influenced by the sound frequency of the audiovisual stimulus and thus by the direction of audiovisual spatial mismatch in the preceding adaptation trial (see Fig. 2), as indicated by a highly significant three-way interaction of Group (750L/3000R or 750R/3000L), Sound Frequency (750 Hz or 3000 Hz) and Adaptation Trial (same or different frequency), *F*(1, 28) = 19.01, *p *< .001. Sub-ANOVAs showed that the two-way interaction of Sound Frequency and Adaptation Trial was significant in both groups (both *p*s ≤ .022). Accordingly, in both groups localization responses differed significantly between the leftward-adapted and the rightward-adapted sound frequency only in same-frequency trials (both *p*s < .01, Bonferroni-corrected), but not in different-frequency trials (both *p*s > .10, Bonferroni-corrected). Moreover, in same frequency-trials the difference between the leftward-adapted and the rightward-adapted sound frequency was also significantly larger than in different-frequency trials (both *p*s < .05, Bonferroni-corrected). The size of this immediate recalibration effect did not vary between locations and/or groups (all *p*s ≥ .133).

Thus, while overall localization responses were shifted in the direction of audiovisual spatial mismatch that was associated with the sound frequency (see Fig. 1), reflecting the cumulative stimulus history, this effect was reduced in unimodal auditory test trials which followed an audiovisual stimulus with a different sound frequency and thus with the opposite direction of spatial mismatch (see Fig. 2). This modulation of the ventriloquism aftereffect reflects the immediate stimulus history and suggests that auditory localization was recalibrated on a trial-by-trial basis as well^12^.

To test for a possible relationship between immediate and cumulative recalibration effects, we correlated the individual values for these two measures. The immediate recalibration effect was calculated as the difference in the size of the aftereffect (i.e., rightward-adapted minus leftward-adapted sound frequency) between same-frequency and different-frequency trials. The cumulative recalibration effect was calculated as the overall difference in localization responses between the leftward-adapted and the rightward-adapted sound frequency across same- and different-frequency trials, as depicted in Fig. 1. The resulting correlation between these two measures was weakly negative (*r *= −.25), but did not reach statistical significance (*p *= .177).

### Accumulation of Recalibration over Trials

**Figure 3:**
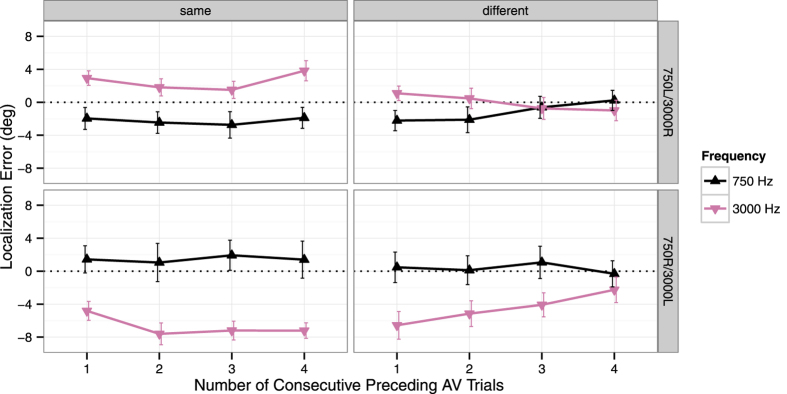
Localization responses in auditory-only trials as a function of the number of consecutive preceding audiovisual (AV) trials with the same or different sound frequency. Mean constant errors (i.e., the mean deviation of the spatial responses from the location of the sound source) across all loudspeaker locations are shown separately for the 750 Hz and 3000 Hz tones. Upper panels show the data for the subgroup of participants (*n *= 15) in which the 750 Hz tone was paired with a visual stimulus to the left and the 3000 Hz tone with a visual stimulus to the right (750L/3000R). Data for the subgroup of participants (*n *= 15) in which the 750 Hz tone was paired with a visual stimulus to the right and the 3000 Hz tone with a visual stimulus to the left (750R/3000L) are shown in the lower panels. Negative values indicate constant errors to the left of the true stimulus location, and positive values indicate constant errors to the right. Error bars denote the *SEM*.

To test whether recalibration to the immediate past accumulated over trials, we additionally analysed the localization responses in unimodal auditory test trials as a function of the number of consecutive audiovisual adaptation trials with one sound frequency (and thus direction of spatial mismatch) in the immediate stimulus history. As seen in Fig. 3, the influence of audiovisual adaptation trials with a sound frequency differing from the unimodal test sound increased over trials: The three-way interaction of Group (750L/3000R or 750R/3000L), Sound Frequency (750 Hz or 3000 Hz) and Consecutive Adaptation Trials (1, 2, 3, or 4) was highly significant, *F*(3, 84) = 13.97, *p *< .001. By contrast, when the sound frequency in the unimodal auditory test trial matched the sound frequency of the preceding adaptation trials, recalibration to the cumulative past [Group x Sound Frequency interaction: *F*(1, 28) = 37.99, *p *< .001] was not further modulated by the number of consecutive adaptation trials in the immediate stimulus history (see Fig. 3), *F *< 1. Thus, an immediate trial-by-trial recalibration, as reported by Wozny and Shams^12^, seems to be mainly triggered when the direction of spatial mismatch conflicts with the cumulative stimulus history.

The results shown in Fig. 3 suggest that within three to four consecutive trials of exposure to a conflicting direction of spatial mismatch in the immediate stimulus history, the cumulative recalibration effect was completely abolished. To make this effect comparable between groups, we calculated the size of the aftereffect (i.e., the difference between leftward-adapted and rightward-adapted sound frequency) for each number of consecutive adaptation trials. The resulting values were submitted to an ANOVA with the between-participants factor Group (750L/3000R or 750R/3000L) and the within-participants factors Adaptation Frequency (same or different) and Consecutive Adaptation Trials (1, 2, 3, or 4). This analysis yielded a highly significant interaction between Adaptation Frequency and Consecutive Adaptation Trials, *F*(3, 84) = 8.48, *p *< .001. This effect did, however, not differ between groups, as neither the main effect nor any interaction involving Group reached statistical significance (all *p*s > .10). Follow-up polynomial contrasts showed that, across groups, the size of the cumulative aftereffect decreased over consecutive different-frequency adaptation trials with a significant linear trend, *F*(1, 28) = 35.92, *p *< .001. By contrast, there was no significant linear, *F*(1, 28) = 2.83, *p *= .104, or higher-order trend, *F*s < 1, over consecutive same-frequency adaptation trials (see Fig. 3).

### Auditory Localization Performance in Comparison to a Naïve Control Group

**Figure 4:**
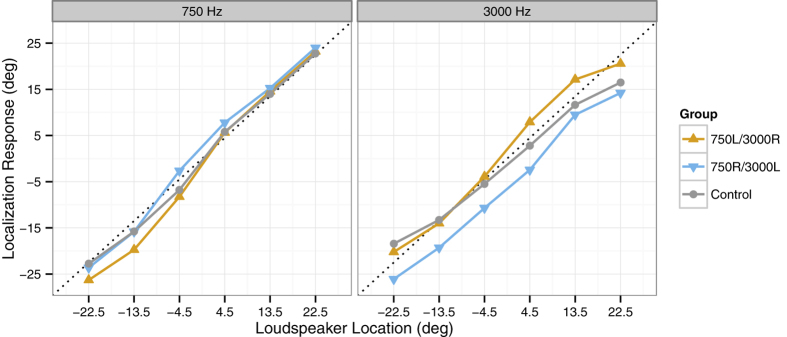
Mean localization responses per loudspeaker location in auditory-only test trials. Localization responses are shown separately for the two experimental groups 750L/3000R (orange) and 750R/3000L (blue), in comparison to a naïve control group (grey) that performed only the unisensory localization task without any audiovisual adaptation. The left panel shows the localization data for the 750 Hz tone, and the right panel shows the localization data for the 3000 Hz tone. Dotted lines indicate the actual locations of the loudspeakers.

Finally, we compared the auditory localization performance of the two experimental groups with a control group of naïve participants (*N *= 26) that performed the unimodal sound localization task without any audiovisual adaptation. The results shown in Fig. 1 suggest that overall there was a slight leftward bias in sound localization responses, relative to the actual loudspeaker locations, in the 750R/3000L group, which was not apparent in the 750L/3000R group. Figure 4 compares the localization responses from these two groups and the control group at each loudspeaker location, separately for the 750 Hz and 3000 Hz tones. As can be seen, for both sound frequencies, localization responses were systematically shifted in opposite directions for the two experimental groups, depending on the adapted direction of audiovisual spatial mismatch, but followed a highly similar pattern across loudspeaker locations. Accordingly, the significant interaction of Group (750L/3000R or 750R/3000L) and Sound Frequency (750 Hz or 3000 Hz) reported above was not further modulated by Location (six levels: ±4.5°, ±13.5° and ±22.5°), *F *< 1. In both groups, however, participants underestimated the eccentricity of the 3000 Hz tones compared to the 750 Hz tones, as reflected by a highly significant interaction between Location and Sound Frequency, *F*(5, 140) = 6.10, *p *< .001. The same effect was observed in the control group that performed the unimodal sound localization task without any audiovisual adaptation (see grey lines in Fig. 4), *F*(5, 125) = 12.33, *p *< .001.

To directly compare localization performance between the control group and the two experimental groups, the data from all three groups was submitted to an ANOVA with the between-participants factor Group (750L/3000R, 750R/3000L, control) and the within-participants factors Location (six levels: ±4.5°, ±13.5° and ±22.5°) and Sound Frequency (750 Hz or 3000 Hz). This analysis again yielded a significant interaction of Location and Sound Frequency, *F*(5, 265) = 16.55, *p *< .001, and a significant interaction of Group and Sound Frequency, *F*(2, 53) = 12.92, *p *< .001. However, neither the main effect of Group, *F*(2, 53) = 1.29, *p *= .285, nor any other interaction involving Group, both *F*s < 1, were significant. Thus, it seems unlikely that the underestimated eccentricity of the 3000 Hz tones, as compared to the 750 Hz tones, was due to the interspersed audiovisual adaptation trials in the experimental groups. Rather, the 3000 Hz tone might have been more difficult to localize than the 750 Hz tone^26^, thus leading to a stronger visual bias^22^ in the two experimental groups. This might explain why the difference between the two experimental groups appeared to be more pronounced for the 3000 Hz tone compared to the 750 Hz tone (see Fig. 4).

## Discussion

In this study, we tested whether or not instantaneous sensory recalibration and recalibration based on accumulated evidence represent the same underlying learning mechanism and, thus, might involve similar or distinct neural systems. In the present paradigm, two sounds, a low- and a high-frequency tone, were paired with opposite directions of audiovisual spatial mismatch (leftward vs. rightward). In accordance with this cumulative stimulus history, localization in unimodal auditory trials was shifted in opposite directions for the two sound frequencies. On a trial-by-trial basis, however, this frequency-specific ventriloquism aftereffect was reduced when the sound was preceded by an audiovisual trial featuring the other sound frequency and direction of spatial mismatch. This immediate recalibration occurred despite the use of a different sound frequency in the audiovisual adaptation and the following auditory test trial.

The results of our new paradigm allowed us to reconcile two main research lines on cross-modal recalibration with conflicting results in the past: First, there has been mixed evidence regarding the sound frequency-specificity of cross-modal recalibration. Some studies reported that recalibration is specific for the sound frequency used during the cross-modal exposure phase, which suggests that representations in early, tonotopically-organized auditory areas are adjusted^3^,^4^,^6^. By contrast, other studies reported complete transfer of cross-modal recalibration across sound frequencies, pointing to an involvement of non-primary and possibly multisensory brain areas^7^,^8^,^27^. Here we show that cross-modal recalibration alters both sound frequency-specific and sound frequency-invariant auditory spatial representations within the same experiment. Second, our results confirm that cross-modal recalibration is triggered on a trial-by-trial basis^11^,^12^, but extend this finding by demonstrating that such immediate adaptations take into account the cumulative stimulus history. Taken together, this pattern of results suggests that sensory recalibration operates at different time scales and seems to involve at least partially distinct neural systems.

The cumulative stimulus history resulted in an independent recalibration of auditory localization for the two sound frequencies in opposite directions. This finding provides strong evidence for a cross-modal adjustment of spatial representations in frequency-specific neural populations, as typical for early auditory cortex^3^,^4^,^5^,^6^. It will be important to determine whether auditory localization can be independently recalibrated based on other stimulus dimensions as well. Recent findings have shown that cross-modal timing perception of asynchronous auditory and visual speech components can recalibrate independently for different speakers (male or female) at the same time^28^. However, this effect might depend on lower-level mechanisms that allow for an independent recalibration at different spatial locations, rather than on higher-level binding of speaker identity, since the speech probes were presented at different spatial locations during the adapation phase^29^. Correspondingly, spatial recalibration in the ventriloquism aftereffect has been shown to be specific for the trained region of space^9^,^30^, but is not modulated by the semantic congruence of the auditory and visual stimuli^31^,^32^. Thus, cross-modal spatial recalibration appears to depend on lower-level sensory representations, in line with the frequency-specificity of the ventriloquism aftereffect that was induced by the cumulative stimulus history in the present study.

The sound frequency-specific adaptation to the cumulative stimulus history appeared to be complete within the first experimental block of around 80 bimodal adaptation trials, as there was no further increase in the magnitude of the ventriloquism aftereffect in subsequent blocks. This fast build-up of cross-modal recalibration is in line with previous studies showing that the maximum strength of recalibration was reached within only one to three minutes of exposure to a consistent audiovisual spatial mismatch^33^. Once acquired, it is assumed that cross-modal recalibration is relatively stable over time and does not dissipate unless the system is confronted with counterevidence^4^,^33^,^34^. Correspondingly, we observed a reduction of the cumulative ventriloquism aftereffect when the direction of audiovisual spatial mismatch in the immediate past conflicted with the cumulative stimulus history. Importantly, however, this immediate recalibration effect was independent of sound frequency and was not significantly correlated with the sound frequency-specific ventriloquism aftereffect induced by the cumulative stimulus history. Thus, although recalibration to the immediate and cumulative past operated at short and partially overlapping time scales, they seem to depend on dissociable underlying mechanisms.

Adaptation to spatially mismatching audiovisual input in adulthood seems to predominantly depend on cortical processing^35^. This is in line with the observation that the influence of spatially mismatching visual stimuli on sound localization in the ventriloquist situation is mediated by pathways running from the visual cortex^36^,^37^ via parietal areas^21^ to the planum temporale in auditory cortex^17^,^19^,^20^. In principle, cross-modally induced changes in sound localization behaviour, as observed in the ventriloquism aftereffect, could be mediated by the same pathway as the online ventriloquist effect, namely feedback influences from multisensory parietal regions on secondary auditory cortex. Alternatively, sensory recalibration in the ventriloquism aftereffect might originate from changes in unisensory auditory representations^38^. The latter hypothesis implies that the online ventriloquist effect and the ventriloquism aftereffect are mediated by dissociable mechanisms, an assumption that is supported by several recent findings. Electrophysiological studies in humans have shown that repeated exposure to audiovisual stimuli with a consistent spatial mismatch affects stages in the auditory cortical processing stream that are earlier than those affected by the online ventriloquist effect^14^. Lesion studies in animals have suggested that recalibration might critically depend even on primary auditory cortical areas^39^. Moreover, it has been shown that the amount of cross-modal recalibration does not depend on the relative reliability of the auditory and visual stimuli during the learning phase^40^, whereas cross-modal integration in the online ventriloquist effect strongly depends on cue reliability^22^. Finally, audiovisual synchrony seems to be a prerequisite for the occurrence of the online ventriloquist effect^25^,^41^, whereas a spatial ventriloquism aftereffect has recently been observed after asynchronous audiovisual stimulation, suggesting that spatial feedback provided by the visual stimuli is the major driver of recalibration^42^.

The available evidence for a dissociation between cross-modal integration and recalibration processes stems from studies that have looked at recalibration following repeated exposure to a consistent cross-modal spatial mismatch^14^,^40^,^42^. The present results extend these findings by showing that recalibration mechanisms following repeated exposure to a consistent cross-modal spatial mismatch are dissociable from instantaneous recalibration mechanisms operating on a trial-by-trial basis and, thus, suggest that both mechanisms are at work in parallel. The immediate sound frequency-invariant adjustment to cross-modal spatial mismatch which we observed on a trial-by-trial basis (see also^12^), similar as the online ventriloquist effect, is likely a result of feedback influences from multisensory parietal structures on auditory cortex^17^,^18^,^19^,^20^,^21^. However, adaptation to the cumulative stimulus history seemed to invoke changes in unisensory auditory spatial representations at an earlier, tonotopically-organized processing stage (see also^3^,^4^,^6^,^14^), possibly as a consequence of a repeated and consistent activation of recalibration via multisensory parietal structures. Thus, cross-modal recalibration might initially affect higher-level multisensory representations and then progress to lower-level modality-specific representations.

A continuous realignment and fine-tuning of modality-specific representations of space might be needed to achieve correct correspondence values across modalities, which would improve individual cue accuracy^40^. This assumption is supported by findings from blind individuals who show, if at all, constant biases in sound localization^43^,^44^, but overall equal or higher sound localization precision^45^. Similarly, in sighted individuals, a temporary absence of visual calibration induced by short-term light deprivation affects mainly sound localization accuracy, rather than localization precision^46^.

The idea that sensory recalibration starts via multisensory representations and ends up with a retuning of sensory maps at earlier processing stages shares similarities with the reverse hierarchy theory of visual perceptual learning^47^. Visual perceptual learning has been defined as a long-term improvement, such as reduced detection or discrimination thresholds, in the ability to perform a visual task that results from training or exposure to a specific visual feature^47^,^48^,^49^. The reverse hierarchy theory states that perceptual learning is a top-down guided process which starts at higher areas of the visual system and then progresses backwards to lower visual areas^47^. For example, after training on an easy visual orientation discrimination task, learning might generalize across orientations and retinal positions, matching the spatial generalization in higher visual areas such as the inferotemporal cortex. With increasing task difficulty, learning becomes more specific to both orientation and retinal position, in line with an involvement of lower visual areas such as V4 or even primary visual cortex^50^. More recently, it has been suggested that learning at a higher cortical stage does not necessarily have to precede learning at lower cortical stages, but that both processes can occur flexibly and even in parallel^51^. Our results similarly show a parallel involvement of higher, sound frequency-invariant, and lower, sound frequency-specific, processing stages in cross-modal sensory recalibration. It might thus be speculated that perceptual learning within one sensory modality and recalibration across sensory modalities involve related underlying learning mechanisms, not disregarding that they represent clearly distinct processes.

In conclusion, our findings show that cross-modal spatial recalibration operates in parallel at different but partially overlapping time scales. While the immediate stimulus history causes an instantaneous, sound frequency-invariant recalibration of sound localization, regularities in the cumulative stimulus history simultaneously result in an adjustment of frequency-specific auditory spatial representations. We propose that instantaneous recalibration and recalibration based on accumulated evidence represent at least partially distinct processes that jointly determine sound localization behaviour.

## Methods

### Participants

Thirty healthy adult volunteers (23 women and seven men), aged from 19 to 47 years (mean 25.2 years), took part in the main experiment. An additional group of 26 adult volunteers (18 women and eight men), aged from 20 to 39 years (mean 26.3 years), performed a unimodal control task. All participants reported normal hearing and normal or corrected-to-normal vision, and all except three participants in the main experiment and one participant in the control experiment were right-handed by self-report. They received course credit or were compensated €7 per hour for their participation. Written informed consent was obtained from all participants prior to taking part. The study was performed in accordance with the ethical standards laid down in the Declaration of Helsinki. The procedure was approved by the ethics commission of the German Psychological Society (DGPs).

### Apparatus

Participants were seated in a dark, sound-attenuated room with their head immobilized by a chin rest. The apparatus consisted of six loudspeakers (ConceptC Satellit, Teufel GmbH, Berlin, Germany) that were located at ear level with eccentricities of 4.5°, 13.5° and 22.5° to the left and to the right side of the participants’ straight-ahead position (0°). The loudspeakers were attached to a semi-circular frame at a distance of 90 cm from the participants’ head position and were hidden from view behind a black, acoustically transparent curtain extending peripherally to 90° from the midline on both sides. For visual stimulation, a red laser pointer was projected onto the curtain at the level of the loudspeakers for 200 ms. The laser pointer was attached to a step motor which allowed stimulation at different azimuthal locations. The auditory stimuli were 750 Hz and 3000 Hz tones with durations of 200 ms (including 5 ms linear rise/fall envelopes), which were presented at 65 dB(A), as measured at the participants’ head position. Stimulus intensity was varied over a 4 dB range for every stimulus presentation to reduce any detectable differences in the loudspeaker transformation functions. Localization responses were made by means of a rotatable hand pointer that was mounted in front of the participant. The hand pointer consisted of a metal rod with a built-in response button. The azimuthal angle of the hand pointer was recorded from a potentiometer whenever the button was pressed.

### Design and Procedure

In each trial of the main experiment, one of the two sound stimuli (750 Hz and 3000 Hz) was presented from one of the six loudspeaker locations, either alone or together with a synchronous but spatially displaced visual stimulus (±13.5° offset from the sound source). Each combination of sound frequency, location, and modality (auditory or audiovisual) was presented 20 times, resulting in 480 trials overall, which were subdivided in three blocks of 160 trials each. Participants were instructed to localize the perceived location of the sound source as accurately as possible in each trial, while ignoring concurrent visual stimuli in audiovisual trials.

Each sound frequency was paired with a constant direction of spatial mismatch in the audiovisual trials. For half of the participants, the 750 Hz tone was always paired with a visual stimulus to the left and the 3000 Hz tone with a visual stimulus to the right, and vice versa for the other half. Auditory and audiovisual trials were presented in a pseudorandom order, with the constraint that for each sound frequency, half of the 120 unimodal auditory trials were preceded by an audiovisual stimulus featuring the same frequency, and half of the trials were preceded by an audiovisual stimulus featuring the other frequency (disregarding any interjacent unimodal auditory trials). Moreover, out of the 60 unimodal trials per sound frequency which were preceded by a same-frequency audiovisual stimulus, in 15 trials each, only the last, the last two, the last three, or the last four audiovisual stimuli featured the same frequency and thus direction of spatial mismatch.

The control experiment was identical to the main experiment, except that only unisensory auditory trials were presented (20 trials per location and sound frequency), resulting in 240 trials overall.

## Additional Information

**How to cite this article**: Bruns, P. and Röder, B. Sensory recalibration integrates information from the immediate and the cumulative past. *Sci. Rep.* **5**, 12739; doi: 10.1038/srep12739 (2015).

## References

[b1] Chen L. & Vroomen J. Intersensory binding across space and time: a tutorial review. Atten. Percept. Psychophys. 75, 790–811 (2013). 23709064 10.3758/s13414-013-0475-4

[b2] Strelnikov K., Rosito M. & Barone P. Effect of audiovisual training on monaural spatial hearing in horizontal plane. PLoS ONE 6, e18344 (2011). 21479241 10.1371/journal.pone.0018344 PMC3066228

[b3] Lewald J. Rapid adaptation to auditory-visual spatial disparity. Learn. Mem. 9, 268–278 (2002). 12359836 10.1101/lm.51402 PMC187125

[b4] Recanzone G. H. Rapidly induced auditory plasticity: the ventriloquism aftereffect. Proc. Natl. Acad. Sci. U.S.A. 95, 869–875 (1998). 9448253 10.1073/pnas.95.3.869 PMC33810

[b5] Recanzone G. H. Interactions of auditory and visual stimuli in space and time. Hear. Res. 258, 89–99 (2009). 19393306 10.1016/j.heares.2009.04.009 PMC2787663

[b6] Woods T. M. & Recanzone G. H. Visually induced plasticity of auditory spatial perception in macaques. Curr. Biol. 14, 1559–1564 (2004). 15341742 10.1016/j.cub.2004.08.059

[b7] Frissen I., Vroomen J., de Gelder B. & Bertelson P. The aftereffects of ventriloquism: are they sound-frequency specific? Acta Psychol. (Amst.) 113, 315–327 (2003). 12835002 10.1016/s0001-6918(03)00043-x

[b8] Frissen I., Vroomen J., de Gelder B. & Bertelson P. The aftereffects of ventriloquism: generalization across sound-frequencies. Acta Psychol. (Amst.) 118, 93–100 (2005). 15627411 10.1016/j.actpsy.2004.10.004

[b9] Kopčo N., Lin I.-F., Shinn-Cunningham B. G. & Groh J. M. Reference frame of the ventriloquism aftereffect. J. Neurosci. 29, 13809–13814 (2009). 19889992 10.1523/JNEUROSCI.2783-09.2009 PMC2804958

[b10] Zwiers M. P., van Opstal A. J. & Paige G. D. Plasticity in human sound localization induced by compressed spatial vision. Nat. Neurosci. 6, 175–181 (2003). 12524547 10.1038/nn999

[b11] Van der Burg E., Alais D. & Cass J. Rapid recalibration to audiovisual asynchrony. J. Neurosci. 33, 14633–14637 (2013). 24027264 10.1523/JNEUROSCI.1182-13.2013 PMC6705173

[b12] Wozny D. R. & Shams L. Recalibration of auditory space following milliseconds of cross-modal discrepancy. J. Neurosci. 31, 4607–4612 (2011). 21430160 10.1523/JNEUROSCI.6079-10.2011 PMC3071751

[b13] Radeau M. & Bertelson P. The after-effects of ventriloquism. Q. J. Exp. Psychol. 26, 63–71 (1974). 4814864 10.1080/14640747408400388

[b14] Bruns P., Liebnau R. & Röder B. Cross-modal training induces changes in spatial representations early in the auditory processing pathway. Psychol. Sci. 22, 1120–1126 (2011). 21771962 10.1177/0956797611416254

[b15] Picton T. W. . Intracerebral sources of human auditory-evoked potentials. Audiol. Neurootol. 4, 64–79 (1999). 9892757 10.1159/000013823

[b16] Verkindt C., Bertrand O., Perrin F., Echallier J.-F. & Pernier J. Tonotopic organization of the human auditory cortex: N100 topography and multiple dipole model analysis. Electroencephalogr. Clin. Neurophysiol. 96, 143–156 (1995). 7535220 10.1016/0168-5597(94)00242-7

[b17] Bonath B. . Neural basis of the ventriloquist illusion. Curr. Biol. 17, 1697–1703 (2007). 17884498 10.1016/j.cub.2007.08.050

[b18] Bruns P. & Röder B. Tactile capture of auditory localization: an event-related potential study. Eur. J. Neurosci. 31, 1844–1857 (2010). 20584189 10.1111/j.1460-9568.2010.07232.x

[b19] Bonath B. . Audio-visual synchrony modulates the ventriloquist illusion and its neural/spatial representation in the auditory cortex. Neuroimage 98, 425–434 (2014). 24814210 10.1016/j.neuroimage.2014.04.077

[b20] Callan A., Callan D. & Ando H. An fMRI study of the ventriloquism effect. Cereb. Cortex (in press). 10.1093/cercor/bhu306 PMC4816779 25577576

[b21] Renzi C. . Spatial remapping in the audio-tactile ventriloquism effect: a TMS investigation on the role of the ventral intraparietal area. J. Cogn. Neurosci. 25, 790–801 (2013). 23363411 10.1162/jocn_a_00362

[b22] Alais D. & Burr D. The ventriloquist effect results from near-optimal bimodal integration. Curr. Biol. 14, 257–262 (2004). 14761661 10.1016/j.cub.2004.01.029

[b23] Bertelson P. & Aschersleben G. Automatic visual bias of perceived auditory location. Psychon. Bull. Rev. 5, 482–489 (1998).

[b24] Bertelson P. & Radeau M. Cross-modal bias and perceptual fusion with auditory-visual spatial discordance. Percept. Psychophys. 29, 578–584 (1981). 7279586 10.3758/bf03207374

[b25] Slutsky D. A. & Recanzone G. H. Temporal and spatial dependency of the ventriloquism effect. Neuroreport 12, 7–10 (2001). 11201094 10.1097/00001756-200101220-00009

[b26] Middlebrooks J. C. & Green D. M. Sound localization by human listeners. Annu. Rev. Psychol. 42, 135–159 (1991). 2018391 10.1146/annurev.ps.42.020191.001031

[b27] Navarra J., Garcia-Morera J. & Spence C. Temporal adaptation to audiovisual asynchrony generalizes across different sound frequencies. Front. Psychol. 3, 152 (2012). 22615705 10.3389/fpsyg.2012.00152 PMC3351678

[b28] Roseboom W. & Arnold D. H. Twice upon a time: multiple concurrent temporal recalibrations of audiovisual speech. Psychol. Sci. 22, 872–877 (2011). 21690312 10.1177/0956797611413293

[b29] Heron J., Roach N. W., Hanson J. V. M, McGraw P. V. & Whitaker D. Audiovisual time perception is spatially specific. Exp. Brain Res. 218, 477–485 (2012). 22367399 10.1007/s00221-012-3038-3 PMC3324684

[b30] Bertelson P., Frissen I., Vroomen J. & de Gelder B. The aftereffects of ventriloquism: patterns of spatial generalization. Percept. Psychophys. 68, 428–436 (2006). 16900834 10.3758/bf03193687

[b31] Radeau M. & Bertelson P. Adaptation to auditory-visual discordance and ventriloquism in semirealistic situations. Percept. Psychophys. 22, 137–146 (1977).

[b32] Radeau M. & Bertelson P. Cognitive factors and adaptation to auditory-visual discordance. Percept. Psychophys. 23, 341–343 (1978). 748857 10.3758/bf03199719

[b33] Frissen I., Vroomen J. & de Gelder B. The aftereffects of ventriloquism: the time course of the visual recalibration of auditory localization. Seeing Perceiving 25, 1–14 (2012). 22353565 10.1163/187847611X620883

[b34] Machulla T.-K., Di Luca M., Froehlich E. & Ernst M. O. Multisensory simultaneity recalibration: storage of the aftereffect in the absence of counterevidence. Exp. Brain Res. 217, 89–97 (2012). 22207361 10.1007/s00221-011-2976-5

[b35] Passamonti C., Frissen I. & Làdavas E. Visual recalibration of auditory spatial perception: two separate neural circuits for perceptual learning. Eur. J. Neurosci. 30, 1141–1150 (2009). 19735289 10.1111/j.1460-9568.2009.06910.x

[b36] Bertini C., Leo F., Avenanti A. & Làdavas E. Independent mechanisms for ventriloquism and multisensory integration as revealed by theta-burst stimulation. Eur. J. Neurosci. 31, 1791–1799 (2010). 20584183 10.1111/j.1460-9568.2010.07200.x

[b37] Leo F., Bolognini N., Passamonti C., Stein B. E. & Làdavas E. Cross-modal localization in hemianopia: new insights on multisensory integration. Brain 131, 855–865 (2008). 18263626 10.1093/brain/awn003

[b38] Shams L. & Seitz A. R. Benefits of multisensory learning. Trends Cogn. Sci. 12, 411–417 (2008). 18805039 10.1016/j.tics.2008.07.006

[b39] Nodal F. R. . Lesions of the auditory cortex impair azimuthal sound localization and its recalibration in ferrets. J. Neurophysiol. 103, 1209–1225 (2010). 20032231 10.1152/jn.00991.2009 PMC2887622

[b40] Zaidel A., Turner A. H. & Angelaki D. E. Multisensory calibration is independent of cue reliability. J. Neurosci. 31, 13949–13962 (2011). 21957256 10.1523/JNEUROSCI.2732-11.2011 PMC3196629

[b41] Lewald J. & Guski R. Cross-modal perceptual integration of spatially and temporally disparate auditory and visual stimuli. Brain Res. Cogn. Brain Res. 16, 468–478 (2003). 12706226 10.1016/s0926-6410(03)00074-0

[b42] Pages D. S. & Groh J. M. Looking at the ventriloquist: visual outcome of eye movements calibrates sound localization. PLoS ONE 8, e72562 (2013). 24009691 10.1371/journal.pone.0072562 PMC3757015

[b43] Lewald J. Opposing effects of head position on sound localization in blind and sighted human subjects. Eur. J. Neurosci. 15, 1219–1224 (2002). 11982632 10.1046/j.1460-9568.2002.01949.x

[b44] Zwiers M. P., van Opstal A. J. & Cruysberg J. R. M. A spatial hearing deficit in early-blind humans. J. Neurosci. 21, RC142 (2001). 11312316 10.1523/JNEUROSCI.21-09-j0002.2001 PMC6762556

[b45] Röder B. . Improved auditory spatial tuning in blind humans. Nature 400, 162–166 (1999). 10408442 10.1038/22106

[b46] Lewald J. More accurate sound localization induced by short-term light deprivation. Neuropsychologia 45, 1215–1222 (2007). 17113113 10.1016/j.neuropsychologia.2006.10.006

[b47] Ahissar M. & Hochstein S. The reverse hierarchy theory of visual perceptual learning. Trends Cogn. Sci. 8, 457–464 (2004). 15450510 10.1016/j.tics.2004.08.011

[b48] Beste C. & Dinse H. R. Learning without training. Curr. Biol. 23, R489–R499 (2013). 23743417 10.1016/j.cub.2013.04.044

[b49] Sasaki Y., Nanez J. E. & Watanabe T. Advances in visual perceptual learning and plasticity. Nat. Rev. Neurosci. 11, 53–60 (2011). 19953104 10.1038/nrn2737 PMC2864603

[b50] Ahissar M. & Hochstein S. Task difficulty and the specificity of perceptual learning. Nature 387, 401–406 (1997). 9163425 10.1038/387401a0

[b51] Shibata K., Sagi D. & Watanabe T. Two-stage model in perceptual learning: toward a unified theory. Ann. N. Y. Acad. Sci. 1316, 18–28 (2014). 24758723 10.1111/nyas.12419 PMC4103699

